# Optimizing Retransmission Threshold in Wireless Sensor Networks

**DOI:** 10.3390/s16050665

**Published:** 2016-05-10

**Authors:** Ran Bi, Yingshu Li, Guozhen Tan, Liang Sun

**Affiliations:** 1 Department of Computer Science, Dalian University of Technology, Dalian 116000, China; gztan@dlut.edu.cn (G.T.); liangsun@dlut.edu.cn (L.S.); 2 Department of Computer Science, Georgia State University, Atlanta, GA 30303, USA; yli@cs.gsu.edu

**Keywords:** packet delivery, optimal retransmission threshold, in time

## Abstract

The retransmission threshold in wireless sensor networks is critical to the latency of data delivery in the networks. However, existing works on data transmission in sensor networks did not consider the optimization of the retransmission threshold, and they simply set the same retransmission threshold for all sensor nodes in advance. The method did not take link quality and delay requirement into account, which decreases the probability of a packet passing its delivery path within a given deadline. This paper investigates the problem of finding optimal retransmission thresholds for relay nodes along a delivery path in a sensor network. The object of optimizing retransmission thresholds is to maximize the summation of the probability of the packet being successfully delivered to the next relay node or destination node in time. A dynamic programming-based distributed algorithm for finding optimal retransmission thresholds for relay nodes along a delivery path in the sensor network is proposed. The time complexity is $O\left(n\Delta \cdot \max_{1\leq i\leq n}\left\{u_{i}\right\}\right)$, where $u_{i}$ is the given upper bound of the retransmission threshold of sensor node *i* in a given delivery path, *n* is the length of the delivery path and Δ is the given upper bound of the transmission delay of the delivery path. If Δ is greater than the polynomial, to reduce the time complexity, a linear programming-based $\left(1+p_{min}\right)$-approximation algorithm is proposed. Furthermore, when the ranges of the upper and lower bounds of retransmission thresholds are big enough, a Lagrange multiplier-based distributed O(1)-approximation algorithm with time complexity O(1) is proposed. Experimental results show that the proposed algorithms have better performance.

## 1. Introduction

Wireless Sensor Networks (WSNs) have been increasingly deployed for a wide variety of real-time applications, such as industrial Internet-of-Things, emergency response, critical infrastructure monitoring and process measurement and control. In real-time applications, deadline misses in data transmission may bring about irreparable damage [1,2,3,4]. For mission-critical tasks, not only the packet delivery deadline should be met, but also the transmission reliability is supposed to be guaranteed. Therefore, providing reliable and timely data delivery in WSNs is crucial to the success of the mission.

In supporting mission critical-tasks, data delivery is required to be timely and reliable, but it is challenging for desirable Quality of Service (QoS). Due to the transmission uncertainties, wireless link dynamics and the queueing dynamics, wireless link qualities in sensor networks can vary at a wide range of timescales [5,6], which also results in node failures and connectivity varying over time [7]. In practical sensor networks, the sensor node may need multiple retransmissions for successfully forwarding a packet at each hop. A maximum number of retransmissions, *i.e.*, retransmission threshold, is usually set for each node to prevent transmitting a packet over a bad link unlimitedly, and the packet will be eventually dropped by the sender after the maximum number of transmission retries. The success of a packet delivery is probabilistic, and thus, the WSNs are rendered unreliable.

QoS supported by the underlying networks aims to meet end users’ satisfaction with the services that the system provides [8]. The QoS provisioning in data delivery can be measured in the form of performance metrics, such as delay, packet loss rate and throughput. There exists a tradeoff among the QoS requirements, such as transmission reliability and deliver delay. Thus, the soft QoS supported can be accomplished, in which the soft QoS refers to achieving the QoS requirements with probability. Many efforts have been made to improve transmission reliability and deliver delay in WSNs, including quality measurement of forwarding data [5,9,10] and real-time routing protocol designing [11,12,13].

**Figure 1:**
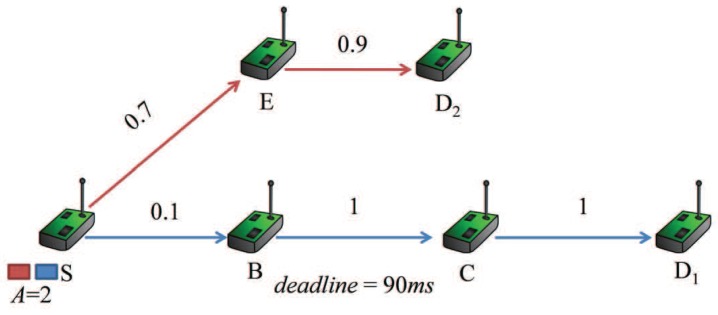
Impact of the retransmission threshold on the probability of packet delivery.

The main methodology applied in the existing works is to exploit a delivery path based on the QoS-aware forwarding quality metrics to improve the performance of packet transmission. However, there exist limited studies on the optimization of the retransmission threshold for each node in a delivery path, which imposes a significant effect on the probability of a packet being successfully transmitted within specified deadline. Most protocols set the same retransmission threshold for all sensor nodes in advance without considering the delay requirement and link quality, so that the delivery performance in terms of latency and reliability is decreased. We use an example inspired by the one in [14] to show the impact of retransmission threshold on the performance, illustrated in Figure 1.

The number associated with each link is the probability for a packet being successfully delivered to the next node through the link, denoted by $p_{suc}$, which means that on average, $1/p_{suc}$ transmission trials are needed to successfully deliver a packet through the link. The integer variable *A* denotes the number of packets in the buffer waiting to be served. The delivery path for the first packet is $S\to B\to C\to D_{1}$, and the path for the second packet is $S\to E\to D_{2}$. We assume that one transmission takes 10 ms, and the deadline of delivering the first packet from node *S* to node $D_{1}$ is 90 ms. For delivering the packet to node $D_{1}$ before the deadline, the summation of transmission trials along the path must be no more than nine, that is the result of 90 ms divided by one transmission time of 10 ms. Traditional protocols set the same retransmission threshold for each relay node in advance. If the protocol is set by a high retransmission threshold, such as 29, in the CTPprotocol [15], without the consideration of delay requirement, thus the probability for successfully transmitting the first packet through the link S→B can be significantly improved. However, this strategy may make the deadlines of both packets expire and consumes more system resource. If the maximum number of transmission trials is three for each node, in view of the summation of transmission trials, there are no more than nine. Then, the probability of the first packet passing the link S→B is $1-0.9^{3}=0.271$. However, if the retransmission thresholds of the three nodes are set to 6, 0 and 0, that is the maximum numbers of transmission trials are 7, 1 and 1, then the probability is $1-0.9^{6}=0.927$. In terms of latency and reliability, there is a tradeoff in determining the retransmission threshold. Therefore, the retransmission threshold should be carefully chosen to achieve desirable QoS provisioning [9].

From the example above, it is obvious that the retransmission threshold has significant impact on the delivery performance in terms of delay and reliability. Few studies focus on the optimization of the retransmission threshold [5,14], and they have the following two major disadvantages. In the feedback control-based framework [5], a heuristic method for adjusting the transmission numbers is proposed, which does not provide the reliability guarantee. In our previous work [14], a greedy-based algorithm for finding optimal retransmission thresholds is proposed, which overlooks the impact of queue length on the forwarding quality. The aforementioned observation motivates us to investigate the problem of finding optimal retransmission thresholds in this paper. The problem is formalized as an integer optimization problem first. Then, a dynamic programming-based distributed algorithm for solving the problem is proposed. It can be run on a sensor node and enables the node to adaptively set the optimal retransmission threshold based on the link quality and the remaining time to deadline. Its time complexity is $O\left(n\Delta \cdot \max_{1\leq i\leq n}\left\{u_{i}\right\}\right)$, where $u_{i}$ is the given upper bound of the retransmission threshold of sensor node *i* in a given delivery path and *n* is the length of the delivery path. The complexity depends on delivery delay Δ. If Δ is less than or equal to the polynomial, the complexity of the algorithm is polynomial. Otherwise, the algorithm is not efficient. In this case, a $\left(1+p_{min}\right)$-approximation algorithm is provided based on the linear programming. Furthermore, when the ranges of the upper and lower bounds of the retransmission thresholds are big enough, a Lagrange multiplier-based distributed O(1)-approximation algorithm with time complexity O(1) is developed. The main contributions of the paper are as follows.

- The problem of finding optimal retransmission thresholds for each node along a delivery path is defined and is formalized as an integer optimization problem.
- A Dynamic Programming-based Distributed Algorithm (DPDA) for solving the problem above is proposed; the correctness of the algorithm is proven; and its time and space complexity are analyzed, *i.e.*, $O\left(n\Delta \cdot \max_{1\leq i\leq n}\left\{u_{i}\right\}\right)$, and $O\left(n\Delta \cdot \max_{1\leq i\leq n}\left\{u_{i}\right\}\right)$.
- In case of the delivery delay Δ being greater than the polynomial, a Linear Programming-based $\left(1+p_{min}\right)$-Approximation Algorithm (LPAA) is proposed.
- Furthermore, in the case of the ranges of the upper and lower bounds of the retransmission thresholds being big enough, a Lagrange Multiplier-based Distributed Approximation Algorithm (LMDAA) with time complexity O(1) is proposed.
- Simulation experiments are conducted to evaluate the proposed algorithms. Simulation results show that the proposed algorithms in the paper have better performance for real-time data delivery.

The rest of this paper is organized as follows. The related works on real-time data delivery are surveyed in Section 2. In Section 3, the problem is described. In Section 4, a dynamic programming-based distributed algorithm for finding optimal retransmission thresholds is provided. Section 5 gives a linear programming-based approximation algorithm, and the ratio bound is analyzed. In Section 6, a Lagrange multiplier-based distributed O(1)-approximation algorithm with time complexity O(1) is proposed. Experimental results are illustrated in Section 7, and Section 8 concludes this paper.

## 2. Related Works

The real-time performance of packet delivery is a fundamental factor in sensor networks, and many efforts have been made to design efficient real-time routing protocols in WSNs. Most of the existing works addressed the following two challenges. First, it is essential to design the metric, which measures the forwarding quality, such as node forwarding quality [9], link quality [10], data aggregation [2] and energy efficiency [12]. Approximate aggregation for tracking quantiles and range countings is presented in [2], where a dynamic binary tree based deterministic tracking algorithm is proposed. Based on the actual number of data transmissions, link-Quality of Forwarding (QoF) and node-QoF metrics are proposed in [9]. A data-driven-based link quality prediction approach is provided in [10], which combines the packet reception ratio and the physical-layer information. Considering queuing delay, the Weighted End-to-End Delay (WEED) metric is introduced in [11]; the work focuses on selecting the path with the minimum expected end-to-end delay. In multi-hop wireless networks, the routing decision takes spatial reusability into account to improve the end-to-end throughput [16]. The authors in [17] utilize the expected available bandwidth to capture the logical intra-flow and inter-flow interference, and an isotonic metric of expected delay is proposed. Dependence among links was revealed recently. By exploiting the low correlated forwarding links, link-correlation Opportunistic Routing (OR) is given in [18]. Facing the uncertainties in wireless communication, the multi-timescale adaptation routing protocol is provided in [6], which can adapt to *in situ* delay conditions in routing delay.

To provide real-time packet delivery, QoS provisioning protocols and transmission scheduling protocols have also been extensively studied. The literature [13] studies geographic opportunistic routing protocol for multi-constrained QoS provisioning in WSNs, and the problem is formulated as a multi-objective multi-constraint optimization problem. By differentiating the priorities of user-level applications, the author in [19] develops an optimization tool for balancing the system resources and presents a cluster-based routing protocol. Under the constraints of delay and load balancing requirements, a centralized heuristic algorithm for selecting the path with minimal cost is provided in [20]. To separate the packet of different QoS requirements, a virtual hybrid potential field is introduced in [21]. Considering capacitated multicast routing problem, the work [22] provides the approximation algorithms for multicast k-path routing and multicast k-tree routing. By supporting slot rescheduling, the segmented slot assignment-based method for improving retransmission efficiency is proposed in [23]. Pan *et al.* [24] formulate joint routing and scheduling into an optimization problem and solve the problem with a column generation method. To route around the dead end relay node, the authors in [25] propose the cross-layer protocol, which integrates geographic routing with the contention-based MAC protocol. The author in [26] map the scheduling of data flow to multiprocessor scheduling and prove an upper bound of the end-to-end communication delay.

There exist limited studies on optimizing the retransmission threshold [5,14]. A distributed route maintenance framework is proposed in [5], which enables the link to adjust the retransmission number. However, the proposed heuristic method cannot provide the reliability guarantee theoretically. In our previous work [14], a greedy-based algorithm for finding optimal retransmission thresholds is proposed, which overlooks the impact of queue length on the forwarding quality. To overcome the shortage of the method, we investigate the problem of finding optimal retransmission thresholds in this paper.

## 3. Problem Description

In this section, the problem of finding optimal retransmission thresholds for each node along a delivery path is defined and is formalized as a general integer optimization problem. We prove that the problem is NP-hard.

### 3.1. Problem Definition

Suppose that the end-to-end path is $P\;=\;j_{1},\;j_{2},\ldots ,\;j_{n+1}$, where $j_{1}$ and $j_{n+1}$ are source and destination nodes and other $j_{i}$’s are relay nodes. There exist the following four attributes for each link $j_{i}\to j_{i+1}$.

- transmission time *t*: This means that once transmission costs time, including encoding and wireless communication. Usually each sensor node takes the same time for once transmission.
- transmission failure probability $p_{i}$: $p_{i}$ denotes the transmission failure probability over the link $j_{i}\to j_{i+1}$. It means that $p_{i}$ is the probability of a transmission failure due to either collisions or bad channel quality when node $j_{i}$ forwards a packet to node $j_{i+1}$.
- retransmission threshold $K_{i}$: This means that the maximum number of retransmission trials is $K_{i}$. It is obvious that the maximum number of transmission retries is $K_{i}+1$, and thus, the packet will be eventually dropped by the sender after the $K_{i}+1$ transmission retries. It can be known that $1-p_{i}^{K_{i}+1}$ is the probability of a packet being successfully delivered to node $j_{i+1}$.
- retransmission threshold $A_{i}$: Each hop delay consists of the transmission delay over the wireless link and the queuing delay in the buffer [9]. $A_{i}$ denotes the number of the packets queued at node $j_{i}$, which means that there exist $A_{i}+1$ packets to be forwarded [6].

We define the metric of a given retransmission threshold as the probability of a packet being successfully delivered through the link. Existing works exploit multipath routing to guarantee both reliability and deliver delay in WSNs [13,27,28]. Similarly to [5,9], we aggregate the measure over a path based on the metric and aim at maximizing the summation of the probability of the packet delivered to the next relay node along the given path within the deadline. The path-metric estimates the given path forwarding quality, and it considers both transmission reliability and delay constraint.

The end-to-end delay over a path is the summation of the delays of all of the hops along the path. Let *δ* denote the delay constraint. Then, the problem of finding the optimal retransmission thresholds for each node can be formulated as the following integer optimization problem, where $L_{i}$ and $U_{i}$ are the given lower and upper bounds of the retransmission threshold of node *i*.

(1) $$\begin{array}{cc} \max & \sum_{i=1}^{n}\left(1-p_{i}^{K_{i}+1}\right)\\ s.t. & \sum_{i=1}^{n}\left(A_{i}+1\right)\left(K_{i}+1\right)t\leq \delta \\ & L_{i}\leq K_{i}\leq U_{i},K_{i}\in Z,i\in \left\{1,2,\ldots ,n\right\} \end{array}$$

Each hop delay consists of the transmission delay and the queuing delay in the buffer; thus, the maximum delay latency for each hop is $\left(A_{i}+1\right)\left(K_{i}+1\right)t$. The first inequality constraint means that the deliver delay of the last packet served at source node $j_{1}$ is no more than the deadline constraint *δ*. The second inequalities mean that the retransmission thresholds should be bounded in a given interval.

For the convenience of formalization and analysis, let Δ denote δ/t. For any i∈{1,2,…,n}, let $a_{i}=A_{i}+1,k_{i}=K_{i}+1,l_{i}=L_{i}+1$ and $u_{i}=U_{i}+1$; then, the problem above is equivalent to the following integer optimization problem.

(2) $$\begin{array}{cc} \min & \sum_{i=1}^{n}p_{i}^{k_{i}}\\ s.t. & \sum_{i=1}^{n}a_{i}k_{i}\leq \Delta \\ & l_{i}\leq k_{i}\leq u_{i},k_{i}\in Z,i\in \left\{1,2,\ldots ,n\right\} \end{array}$$

### 3.2. Computational Complexity Analysis

In this subsection, we construct a *0-1 Knapsack problem* [29], and we prove that integer optimization Equation (2) is equivalent to the proposed *0-1 Knapsack problem*. The computational complexity of the *0-1 Knapsack problem* is NP-hard [29]; thus the hardness of the problem for calculating optimal retransmission thresholds is NP-hard.

Let $S_{i,j}$ denote $p_{i}^{l_{i}+j}-p_{i}^{l_{i}+j-1}$, where $j\in \{1,2,\ldots ,u_{i}-l_{i}\}$. It can be known that $p_{i}^{l_{i}+j}=\sum_{k=1}^{j}S_{i,k}+p_{i}^{l_{i}}$. Thus, we can formulate a 0-1 Knapsack problem as follows.

(3) $$\begin{array}{cc} \min & \sum_{i=1}^{n}\sum_{j=1}^{u_{i}-l_{i}}C_{i,j}S_{i,j}+p_{i}^{l_{i}}\\ s.t. & \sum_{i=1}^{n}\sum_{j=1}^{u_{i}-l_{i}}a_{i}C_{i,j}+\sum_{i=1}^{n}a_{i}l_{i}\leq \Delta \\ & C_{i,j}\in \left\{0,1\right\},i\in \left\{1,2,\ldots ,n\right\},j\in \left\{1,\ldots ,u_{i}-l_{i}\right\} \end{array}$$

**Lemma** **1.**  *For* ∀i∈{1,…,n}, *the following inequalities hold if* $0<p_{i}<1$ *and* $l_{i}<u_{i}$,

(4) $$p_{i}^{l_{i}+j}-p_{i}^{l_{i}+j-1}<p_{i}^{l_{i}+j+1}-p_{i}^{l_{i}+j}<0$$

*where* $j\in \{1,\ldots ,u_{i}-l_{i}\}$.

**Proof.**  Let $g_{i}\left(x\right)=p_{i}^{x}$. For any i∈{1,…,n}, it is obvious that $g_{i}\left(x\right)$ is a monotone decreasing function; thus, $p_{i}^{l_{i}+j+1}-p_{i}^{l_{i}+j}<0$. Obviously, $p_{i}\left(p_{i}^{l_{i}+j}-p_{i}^{l_{i}+j-1}\right)=\left(p_{i}^{l_{i}+j+1}-p_{i}^{l_{i}+j}\right)$. Since $0<p_{i}<1$ and $p_{i}^{l_{i}+j+1}-p_{i}^{l_{i}+j}<0$, we have $p_{i}^{l_{i}+j}-p_{i}^{l_{i}+j-1}<p_{i}^{l_{i}+j+1}-p_{i}^{l_{i}+j}$. In conclusion, $p_{i}^{l_{i}+j}-p_{i}^{l_{i}+j-1}<p_{i}^{l_{i}+j+1}-p_{i}^{l_{i}+j}<0$. ☐

**Lemma** **2.**  *If* $C_{1,1}^{*},C_{1,2}^{*},\ldots ,C_{1,u_{1}-l_{1}}^{*},C_{2,1}^{*},\ldots ,C_{n,u_{n}-l_{n}}^{*}$ *are the optimal solutions to Equation (3), then there exist* $j_{1},\ldots ,j_{n}$, *such that:*

(5) $$C_{i,k}^{*}=\left\{\begin{array}{cc} 1 & k\leq j_{i}\\ 0 & k>j_{i} \end{array}\right.$$

*where* $i\in \left\{1,2,\ldots ,n\right\},k\in \left\{1,\ldots ,u_{i}-l_{i}\right\},j_{i}\in \left\{0,1,\ldots ,u_{i}-l_{i}\right\}$.

**Proof.**  The proof is by contradiction. Suppose that $C_{1,1}^{*},C_{1,2}^{*},\ldots ,C_{1,u_{1}-l_{1}}^{*},C_{2,1}^{*},\ldots ,C_{n,u_{n}-l_{n}}^{*}$ are the optimal solutions to Equation (3) and that they enable the objective function value to achieve the minimum. For given *m*, if there is no integer in $\left\{0,1,\ldots ,u_{m}-l_{m}\right\}$ such that Equation (5) are true, then there must exist integers $r,t,q\in \{1,\ldots ,u_{m}-l_{m}\}$ such that $C_{m,r}^{*}=1,C_{m,t}^{*}=0,C_{m,q}^{*}=1$ and r<t<q. We can construct a solution, whose objective function values are less than that of $C_{1,1}^{*},\ldots ,C_{n,u_{n}-l_{n}}^{*}$, by exchanging $C_{m,t}^{*}$ and $C_{m,q}^{*}$. Suppose that $C_{1,1}^{\prime },C_{1,2}^{\prime },\ldots ,C_{n,u_{n}-l_{n}}^{\prime }$ are derived by merely exchanging $C_{m,t}^{*}$ and $C_{m,q}^{*}$.

Firstly, we prove that $C_{1,1}^{\prime },C_{1,2}^{\prime },\ldots ,C_{n,u_{n}-l_{n}}^{\prime }$ are feasible solutions of Equation (3). For any i∈{1,2,…,n} and $j\in \{1,\ldots ,u_{i}-l_{i}\}$, $C_{i,j}^{*}$ and $C_{i,j}^{\prime }$ are 0,1 variables. It is obvious that $\sum_{j=1}^{u_{m}-l_{m}}a_{m}C_{m,j}^{*}=\sum_{j=1}^{u_{m}-l_{m}}a_{m}C_{m,j}^{\prime }$. According to the construction of the solution, $C_{i,j}^{*}$ and $C_{i,j}^{\prime }$ are identical correspondingly, except for $C_{m,t}^{*}$ and $C_{m,q}^{*}$. Since $\sum_{i=1}^{n}\sum_{j=1}^{u_{i}-l_{i}}a_{i}C_{i,j}^{*}+\sum_{i=1}^{n}a_{i}l_{i}\leq \Delta$, we have $\sum_{i=1}^{n}\sum_{j=1}^{u_{i}-l_{i}}a_{i}C_{i,j}^{\prime }+\sum_{i=1}^{n}a_{i}l_{i}\leq \Delta$. Thus, $C_{1,1}^{\prime },C_{1,2}^{\prime },\ldots ,C_{n,u_{n}-l_{n}}^{\prime }$ are feasible solutions.

Let $\varepsilon =\sum_{i=1}^{n}\sum_{j=1}^{u_{i}-l_{i}}C_{i,j}^{*}S_{i,j}+p_{i}^{l_{i}}-\sum_{i=1}^{n}\sum_{j=1}^{u_{i}-l_{i}}C_{i,j}^{\prime }S_{i,j}+p_{i}^{l_{i}}$ be the difference between the values of the two objective functions. It is easily derived that $\varepsilon =\sum_{j=1}^{u_{m}-l_{m}}C_{m,j}^{*}S_{m,j}-\sum_{j=1}^{u_{m}-l_{m}}C_{m,j}^{\prime }S_{m,j}=S_{m,q}-S_{m,t}$. From Lemma 1, we know ε>0. Then, $C_{1,1}^{\prime },\ldots ,C_{n,u_{n}-l_{n}}^{\prime }$ are more optimal solutions, and it contradicts the fact that $C_{1,1}^{*},\ldots ,C_{n,u_{n}-l_{n}}^{*}$ are the optimal solutions. ☐

**Theorem** **3.**  *Suppose that* $C_{1,1}^{*},\ldots ,C_{n,u_{n}-l_{n}}^{*}$ *are the optimal solutions to Equation (3). For any* i∈{1,2,…,n}, *let* $k_{i}^{*}=\sum_{j=1}^{u_{i}-l_{i}}C_{i,j}^{*}+l_{i}$. *Then,* $k_{1}^{*},\ldots ,k_{n}^{*}$ *are the optimal solutions to Equation (2), and the values of the two objective functions are identical.*

**Proof.**  Suppose that $k_{1}^{\prime },\ldots ,k_{n}^{\prime }$ are the optimal solutions of Equation (2), then we can construct feasible solutions $C_{1,1}^{\prime },\ldots ,C_{n,u_{n}-l_{n}}^{\prime }$ to Equation (3) as following, where $i\in \left\{1,2,\ldots ,n\right\},j\in \left\{1,\ldots ,u_{i}-l_{i}\right\}$.

(6) $$C_{i,j}^{\prime }=\left\{\begin{array}{cc} 1 & j\leq k_{i}^{\prime }-l_{i}\\ 0 & j>k_{i}^{\prime }-l \end{array}\right.$$

It can be known that $\sum_{i=1}^{n}\sum_{j=1}^{u_{i}-l_{i}}a_{i}C_{i,j}^{\prime }+\sum_{i=1}^{n}a_{i}l_{i}=\sum_{i=1}^{n}\sum_{j=1}^{k_{i}^{\prime }-l_{i}}a_{i}C_{i,j}^{\prime }+\sum_{i=1}^{n}a_{i}l_{i}=\sum_{i=1}^{n}a_{i}\left(k_{i}^{\prime }-l_{i}\right)+\sum_{i=1}^{n}a_{i}l_{i}=\sum_{i=1}^{n}a_{i}k_{i}^{\prime }$. Since $\sum_{i=1}^{n}a_{i}k_{i}^{\prime }\leq \Delta$, $C_{1,1}^{\prime },\ldots ,C_{n,u_{n}-l_{n}}^{\prime }$ are the feasible solutions to Equation (3). Based on the definition of $S_{i,j}$, it can be derived that $\sum_{i=1}^{n}p_{i}^{l_{i}+k_{i}^{\prime }-l_{i}}=\sum_{i=1}^{n}\sum_{j=1}^{k_{i}^{\prime }-l_{i}}S_{i,j}+p_{i}^{l_{i}}=\sum_{i=1}^{n}\sum_{j=1}^{k_{i}^{\prime }-l_{i}}C_{i,j}^{\prime }S_{i,j}+p_{i}^{l_{i}}=\sum_{i=1}^{n}\sum_{j=1}^{u_{i}-l_{i}}C_{i,j}^{\prime }S_{i,j}+p_{i}^{l_{i}}$. Thus, the following inequality can be derived.

$$\min\sum_{i=1}^{n}p_{i}^{k_{i}}=\sum_{i=1}^{n}p_{i}^{k_{i}^{\prime }}=\sum_{i=1}^{n}\sum_{j=1}^{u_{i}-l_{i}}C_{i,j}^{\prime }S_{i,j}+p_{i}^{l_{i}}\geq \min\sum_{i=1}^{n}\sum_{j=1}^{u_{i}-l_{i}}C_{i,j}S_{i,j}+p_{i}^{l_{i}}$$

Therefore, the objective function value of Equation (3) is a lower bound of that of Equation (2).

Since $C_{1,1}^{*},\ldots ,C_{n,u_{n}-l_{n}}^{*}$ are the optimal solutions of Equation (3), there exist $j_{1},\ldots ,j_{n}$, such that Equation (5) hold according to Lemma 2. For any *i*, $\sum_{j=1}^{u_{i}-l_{i}}C_{i,j}^{*}=\sum_{j=1}^{j_{i}}C_{i,j}^{*}=j_{i}$ from Equation (5), then $\sum_{j=1}^{u_{i}-l_{i}}C_{i,j}^{*}S_{i,j}+p_{i}^{l_{i}}=\sum_{j=1}^{j_{i}}S_{i,j}+p_{i}^{l_{i}}=p_{i}^{l_{i}+j_{i}}$. Since $k_{i}^{*}=\sum_{j=1}^{u_{i}-l_{i}}C_{i,j}^{*}+l_{i}=j_{i}+l_{i}$, we have $\sum_{i=1}^{n}\sum_{j=1}^{u_{i}-l_{i}}a_{i}C_{i,j}^{*}+\sum_{i=1}^{n}a_{i}l_{i}=\sum_{i=1}^{n}\sum_{j=1}^{j_{i}}a_{i}C_{i,j}^{*}+\sum_{i=1}^{n}a_{i}l_{i}=\sum_{i=1}^{n}a_{i}j_{i}+\sum_{i=1}^{n}a_{i}l_{i}=\sum_{i=1}^{n}a_{i}\left(l_{i}+j_{i}\right)=\sum_{i=1}^{n}a_{i}k_{i}^{*}$. Since $C_{1,1}^{*},\ldots ,C_{n,u_{n}-l_{n}}^{*}$ satisfy all of the constraints of Equation (3), $\sum_{i=1}^{n}a_{i}k_{i}^{*}\leq \Delta$. For any *i*, it is obvious that $0\leq j_{i}\leq u_{i}-l_{i}$. Thus, $k_{1}^{*},\ldots ,k_{n}^{*}$ are the feasible solutions of Equation (2), and hence:

(7) $$\begin{array}{cc} \text{min}\sum_{i=1}^{n}\sum_{j=1}^{u_{i}-l_{i}}C_{i,j}S_{i,j}+p_{i}^{l_{i}} & =\sum_{i=1}^{n}\sum_{j=1}^{u_{i}-l_{i}}C_{i,j}^{*}S_{i,j}+p_{i}^{l_{i}}=\sum_{i=1}^{n}p_{i}^{k_{i}^{*}}\\ & \geq \min\sum_{i=1}^{n}p_{i}^{k_{i}} \end{array}$$

Thus, the objective function value of Equation (2) is a lower bound of that of Equation (3). Based on the analysis above, we can conclude that $k_{1}^{*},\ldots ,k_{n}^{*}$ are the optimal solutions of Equation (2); the values of the two objective functions are identical. ☐

**Corollary** **4.**  *The hardness of integer optimization Equation (2) is* NP-hard. *That is, finding optimal retransmission thresholds for each node in a delivery path is* NP-hard.

**Proof.**  Based on Theorem 3, the hardness of Equation (2) is identical to 0-1 programming Equation (3), which is equivalent to the *Knapsack problem*. Since the *Knapsack problem* is *NP-hard* [29], the hardness of integer optimization Equation (2) is *NP-hard*. ☐

## 4. Dynamic Programming-Based Distributed Algorithm for Optimal Retransmission Thresholds

As analyzed in Section 3.2, the problem of finding optimal retransmission thresholds is equivalent to the *0-1 Knapsack Problem*. Thus, it can be solved by a dynamic programming-based pseudo-polynomial time algorithm [29]. In this section, we first prove the correctness of the dynamic programming-based algorithm, which can output the optimal resolutions to Equation (3). We design a dynamic programming-based algorithm to solve the proposed *0-1 Knapsack Problem*, and then, according to Theorem 3, the optimal retransmission thresholds can be derived based on the resolutions to Equation (3).

**Theorem** **5.**  *If* $C_{1,1}^{\prime },C_{1,2}^{\prime },\ldots ,C_{n,u_{n}-l_{n}}^{\prime }$ *are the optimal solutions to Equation (3), then* $C_{1,2}^{\prime },\ldots ,C_{n,u_{n}-l_{n}}^{\prime }$ *are the optimal solutions to the following problem.*

(8) $$\begin{array}{cc} \text{min} & \sum_{i=2}^{n}\sum_{j=1}^{u_{i}-l_{i}}C_{i,j}S_{i,j}+p_{i}^{l_{i}}+\sum_{j=2}^{u_{1}-l_{1}}C_{1,j}S_{1,j}+p_{1}^{l_{1}}\\ s.t. & \sum_{i=2}^{n}\sum_{j=1}^{u_{i}-l_{i}}a_{i}C_{i,j}+\sum_{j=2}^{u_{1}-l_{1}}a_{1}C_{1,j}+\sum_{i=1}^{n}a_{i}l_{i}\leq \Delta -a_{1}C_{1,1}^{\prime }\\ & C_{i,j}\in \left\{0,1\right\},j\in \left\{1,\ldots ,u_{i}-l_{i}\right\},i\in \left\{1,2,\ldots ,n\right\} \end{array}$$

**Proof.**  The proof is by contradiction. If $C_{1,2}^{\prime },\ldots ,C_{n,u_{n}-l_{n}}^{\prime }$ are not optimal solutions to Equation (8), suppose that $Z_{1,2}^{\prime },\ldots ,Z_{n,u_{n}-l_{n}}^{\prime }$ are the optimal solutions of Equation (8). Then, $C_{1,1}^{\prime },Z_{1,2}^{\prime },\ldots ,Z_{n,u_{n}-l_{n}}^{\prime }$ are batter solutions of Equation (3), which leads to a contradiction. The proof demonstrates the correctness of the dynamic programming-based algorithm. ☐

To briefly describe the recursive procedure, based on Lemma 2, the following mappings are introduced,

$$\begin{array}{c} f:N\times N\to N,f\left(i,j\right)=\sum_{m=0}^{i-1}\left(u_{m}-l_{m}\right)+j\\ g:N\to N,g\left(h\right)=\min\left\{i|h-\sum_{m=0}^{i}\left(u_{m}-l_{m}\right)\leq 0,i\geq 1\right\} \end{array}$$

where $u_{0}=l_{0}=0$. Let $C_{i,j}=C_{f(i,j)},S_{i,j}=S_{f(i,j)}$ and $sum=\sum_{m=1}^{n}\left(u_{m}-l_{m}\right)$, then Equation (3) is equivalent to the following knapsack problem.

(9) $$\begin{array}{cc} \text{min} & \sum_{h=1}^{sum}C_{h}S_{h}+\sum_{i=1}^{n}p_{i}^{l_{i}}\\ s.t. & \sum_{h=1}^{sum}a_{g(h)}C_{h}+\sum_{i=1}^{n}a_{i}l_{i}\leq \Delta \\ & C_{h}\in \left\{0,1\right\},h\in \left\{1,2,\ldots ,sum\right\} \end{array}$$

When the object set is $\left\{S_{h},S_{h+1},\ldots ,S_{sum}\right\}$ and the capacity is bounded by *j*, we use m(h,j) to express the minimum cost. Then, we derive the recursive equation of m(h,j).

$$m\left(h,j\right)=\left\{\begin{array}{cc} m(h+1,j), & 0\leq j-\sum_{h=1}^{n}a_{h}l_{h}<a_{g(h)}\\ \min\left\{m\left(h+1,j\right),m\left(h+1,j-a_{g(h)}\right)+S_{h}\right\}, & j-\sum_{h=1}^{n}a_{h}l_{h}\geq a_{g(h)} \end{array}\right.$$

Additionally, the initial conditions are as follows.

$$m\left(sum,j\right)=\left\{\begin{array}{cc} 0 & 0\leq j-\sum_{h=1}^{n}a_{h}l_{h}<a_{n}\\ S_{sum} & j-\sum_{h=1}^{n}a_{h}l_{h}\geq a_{n} \end{array}\right.$$

Suppose that node $j_{i}$ needs to forward a packet with delivery delay $\Delta^{\prime }$ and that the end-to-end path is $j_{i},j_{i+1},\ldots ,j_{n}$. Since the packet is the first one queued at node $j_{i}$, $a_{i}=1$. Therefore, finding the optimal retransmission threshold $k_{i}$ for the packet over link from $j_{i}$ to $j_{i+1}$ is equivalent to solving the following integer optimization problem.

(10) $$\begin{array}{cc} \text{min} & \sum_{v=i}^{n}p_{v}^{k_{v}}\\ s.t. & \sum_{v=i+1}^{n}a_{v}k_{v}+k_{i}\leq \Delta^{\prime }\\ & l_{v}\leq k_{v}\leq u_{v},k_{v}\in Z,v\in \left\{i,i+1,\ldots ,n\right\} \end{array}$$

According to Theorem 3, the above Equation (10) is equivalent to the following knapsack Equation (11).

(11) $$\begin{array}{cc} \text{min} & \sum_{h=u_{i-1}-l_{i-1}+1}^{sum}C_{h}S_{h}+\sum_{v=i}^{n}p_{v}^{l_{v}}\\ s.t. & \sum_{h=u_{i-1}-l_{i-1}+1}^{sum}a_{g(h)}C_{h}+\sum_{v=i}^{n}a_{v}l_{v}\leq \Delta^{\prime }\\ & C_{h}\in \left\{0,1\right\},h\in \left\{u_{i-1}-l_{i-1}+1,\ldots ,sum\right\} \end{array}$$

Based on the above analysis, knapsack Equation (11) is a special instance of Equation (9), where the `object’ set is $\left\{S_{u_{i-1}-l_{i-1}+1},\ldots ,S_{sum}\right\}$ and the `capacity’ is bounded by $\Delta^{\prime }$. Suppose $C_{u_{i-1}-l_{i-1}+1}^{\prime },\ldots .,C_{sum}^{\prime }$ are the optimal solutions of Equation (11), then $\sum_{h=u_{i-1}-l_{i-1}+1}^{u_{i}-l_{i}}C_{h}^{\prime }$ is the optimal retransmission threshold of node $j_{i}$. The message exchanges among network nodes are enhanced to carry necessary information of link quality and queue length [6,11], so that each node can independently calculate the optimal retransmission threshold based on the path forwarding quality and the remaining time to deadline. Such a property enables the node to adaptively set the optimal retransmission threshold in a scalable manner. The proposed dynamic-based distributed algorithm for the optimal retransmission threshold is described in Algorithm 1.

We present the analysis for the computational complexity of Algorithm 1. The recurrence process of the algorithm yields the time computation of $\left(\Delta^{\prime }-\sum_{h=i}^{n}a_{i}l_{i}\right)\cdot \left(\sum_{h=i}^{n}u_{i}-l_{i}\right)$. Thus, the time complexity of the proposed distributed algorithm is $O\left(n\Delta \cdot \max_{1\leq i\leq n}\left\{u_{i}\right\}\right)$. Space complexity can be similarly analyzed, that is $O\left(n\Delta \cdot \max_{1\leq i\leq n}\left\{u_{i}\right\}\right)$.

## 5. Linear Programming-Based Approximation Algorithm

The dynamic programming-based algorithm proposed in Section 4 is not efficient, if Δ is greater than the polynomial function with respect to *n*. In this section, we construct a linear programming problem and prove that the solution of the proposed problem can be used to construct the approximate solution of integer optimization Equation (2). Additionally, then, a linear programming-based $\left(1+p_{min}\right)$-approximation algorithm is provided.

### 5.1. Mathematical Foundations

**Figure 2:**
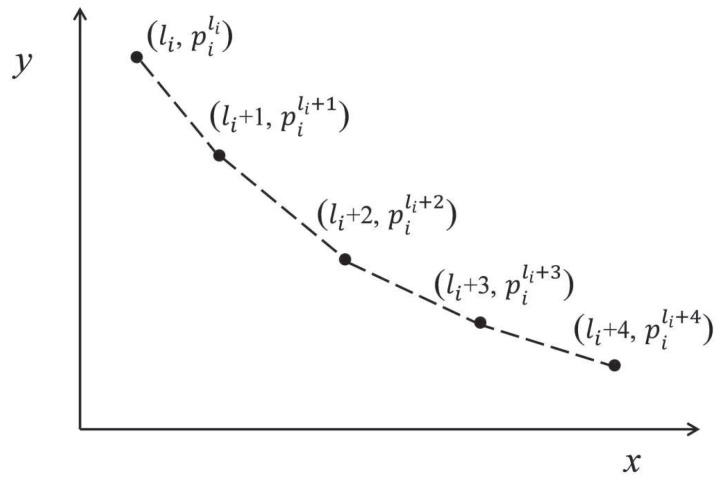
Example of a piecewise linear function.

According to $p_{i},l_{i}$ and $u_{i}$, piecewise linear function $f_{i}\left(z\right)$ is defined as follows, where $h\;\in \;\{0,1,\ldots ,u_{i}-l_{i}-1\}$. We use an example to illustrate the defined function, as depicted in Figure 2.

$$f_{i}\left(z\right)=\left\{\begin{array}{cc} \left(z-l_{i}-h\right)\left(p_{i}^{l_{i}+h+1}-p_{i}^{l_{i}+h}\right)+p_{i}^{l_{i}+h}, & \;z\in (l_{i}+h,l_{i}+h+1)\\ p_{i}^{z}, & z\in \{l_{i},l_{i}+1,\ldots ,u_{i}\} \end{array}\right.$$

**Lemma** **6.**  *For any* i∈{1,2,…,n}, $f_{i}\left(z\right)$ *is a convex function.*

**Algorithm 1:** Dynamic Programming-based Distributed Algorithm for the optimal retransmission threshold (DPDA). 
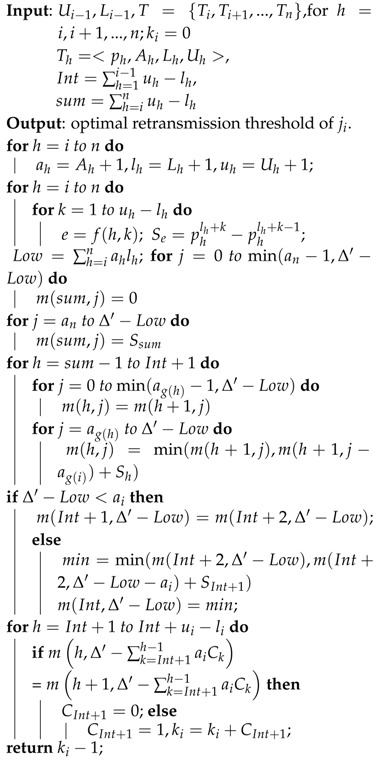

**Proof.**  For any *i*, since $f_{i}\left(z\right)$ is a piecewise linear function, $f_{i}^{\prime \prime }\left(z\right)=0$, where $z\in (l_{i}+h,l_{i}+h+1)$ and $h\in \{0,1,\ldots ,u_{i}-l_{i}-1\}$. Thus, $f_{i}\left(z\right)$ satisfies the sufficient condition of convexity function if $z\in (l_{i}+h,l_{i}+h+1)$. Now, we prove that $f_{i}\left(z\right)$ is a convex function if $z\in \{l_{i},l_{i}+1,\ldots ,u_{i}\}$.

Suppose that $z=\sum_{h=1}^{n}\lambda_{h}z_{h}$, where $z\in \{l_{i},l_{i}+1,\ldots ,u_{i}\},\lambda_{1},\ldots ,\lambda_{n}\in \left[0,1\right],z_{1},\ldots ,z_{n}\in \left[l_{i},u_{i}\right]$ and $\sum_{h=1}^{n}\lambda_{h}=1$. Since ${\left(p_{i}^{x}\right)}^{\prime \prime }>0$, $p_{i}^{x}$ is a convex function. Thus, $p_{i}^{z}\leq \sum_{h=1}^{n}\lambda_{h}p_{i}^{z_{h}}$. For any $z_{h}$, there must exist $j_{h}\in \left\{0,1,\ldots ,u_{i}-l_{i}-1\right\}$, such that $z_{h}\in \left[l_{i}+j_{h},l_{i}+j_{h}+1\right]$.

From the definition of $f_{i}\left(z\right)$, $f_{i}\left(z_{h}\right)=\left(z_{h}-l_{i}-j_{h}\right)\left(p_{i}^{l_{i}+j_{h}+1}-p_{i}^{l_{i}+j_{h}}\right)+p_{i}^{l_{i}+j_{h}}$. Let $b_{i,z_{h}}^{\prime }=1+l_{i}+j_{h}-z_{h},b_{i,z_{h}}^{\prime \prime }=z_{h}-l_{i}-j_{h}$, we have $f_{i}\left(z_{h}\right)=b_{i,z_{h}}^{\prime }p_{i}^{l_{i}+j_{h}}+b_{i,z_{h}}^{\prime \prime }p_{i}^{l_{i}+j_{h}+1}$, $b_{i,z_{h}}^{\prime },b_{i,z_{h}}^{\prime \prime }\in \left[0,1\right]$ and $b_{i,z_{h}}^{\prime }\;+\;b_{i,z_{h}}^{\prime \prime }\;=\;1$. Since $z_{h}\;=b_{i,z_{h}}^{\prime }\left(l_{i}+j_{h}\right)+b_{i,z_{h}}^{\prime \prime }\left(l_{i}+j_{h}+1\right)$, then $p_{i}^{z_{h}}\leq f_{i}\left(z_{h}\right)$ according to the convexity of $p_{i}^{x}$. Based on the above analysis, it can be derived that $p_{i}^{z}\leq \sum_{h=1}^{n}\lambda_{h}p_{i}^{z_{h}}\leq \sum_{h=1}^{n}\lambda_{h}f_{i}\left(z_{h}\right)$. In conclusion, for any i∈{1,2,…,n}, $f_{i}\left(z\right)$ is a convex function. ☐

According to the constraints of Equation (2), a general optimization problem can be formulated as follows.

(12) $$\begin{array}{cc} \min & \sum_{i=1}^{n}f_{i}\left(z_{i}\right)\\ s.t. & \sum_{i=1}^{n}a_{i}z_{i}\leq \Delta \\ & l_{i}\leq z_{i}\leq u_{i},,i\in \left\{1,2,\ldots ,n\right\} \end{array}$$

**Theorem** **7.**  The objective function value of Equation (12) is a lower bound of that of Equation (2), which implies that a feasible solution can be derived based on the optimal resolutions of Equation (12).

**Proof.**  Suppose that $k_{1}^{\prime },k_{2}^{\prime },\ldots ,k_{n}^{\prime }$ are the optimal solutions to Equation (2). For any i∈{1,2,…,n}, let $x_{i}^{\prime }=k_{i}^{\prime }$. Thus, $x_{1}^{\prime },x_{2}^{\prime },\ldots ,x_{n}^{\prime }$ are feasible solutions to Equation (12), and hence:

$$\text{min}\sum_{i=1}^{n}p_{i}^{k_{i}}=\sum_{i=1}^{n}p_{i}^{k_{i}^{\prime }}=\sum_{i=1}^{n}f_{i}\left(x_{i}^{\prime }\right)\geq \min\sum_{i=1}^{n}f_{i}\left(z_{i}\right)$$

Therefore, the objective function value of Equation (12) is a lower bound of that of Equation (2). ☐

**Theorem** **8.**  *The proposed optimization Equation (12) is equivalent to the following linear programming problem, and then, the general optimization Equation (12) can be solved by the linear programming technique.*

(13) $$\begin{array}{cc} \min & \sum_{i=1}^{n}\sum_{j=0}^{u_{i}-l_{i}}\lambda_{i,j}p_{i}^{l_{i}+j}\\ s.t. & \sum_{i=1}^{n}a_{i}\sum_{j=0}^{u_{i}-l_{i}}\lambda_{i,j}\left(l_{i}+j\right)\leq \Delta \\ & \sum_{j=0}^{u_{i}-l_{i}}\lambda_{i,j}=1,0\leq \lambda_{i,j}\leq 1,\\ & j\in \left\{0,\ldots ,u_{i}-l_{i}\right\},i\in \left\{1,2,\ldots ,n\right\} \end{array}$$

**Proof.**  For any i∈{1,2,…,n}, we have $l_{i}\leq \sum_{j=0}^{u_{i}-l_{i}}\lambda_{i,j}\left(l_{i}+j\right)\leq u_{i}$. It is easily known that for any $z_{i}\in \left[l_{i},u_{i}\right]$, there exist $\lambda_{i,0},\ldots ,\lambda_{i,u_{i}-l_{i}}\in \left[0,1\right]$, such that $z_{i}=\sum_{j=0}^{u_{i}-l_{i}}\lambda_{i,j}\left(l_{i}+j\right)$ and $\sum_{j=0}^{u_{i}-l_{i}}\lambda_{i,j}=1$. Additionally, similarly for any $\lambda_{i,0},\ldots ,\lambda_{i,u_{i}-l_{i}}\in \left[0,1\right]$, if $\sum_{j=0}^{u_{i}-l_{i}}\lambda_{i,j}=1$, there must exist $z_{i}\in \left[l_{i},u_{i}\right]$, such that $z_{i}=\sum_{j=0}^{u_{i}-l_{i}}\lambda_{i,j}\left(l_{i}+j\right)$.

Suppose that $\lambda_{1,0},\lambda_{1,1},\ldots ,\lambda_{2,0},\ldots ,\lambda_{n,u_{n}-l_{n}}$ are feasible solutions of Equation (13). For any i∈{1,2,…,n}, let $z_{i}=\sum_{j=0}^{u_{i}-l_{i}}\lambda_{i,j}\left(l_{i}+j\right)$. Thus, $z_{1},\ldots ,z_{n}$ are feasible solutions of Equation (12). According to the convexity of $f_{i}\left(z\right)$, we have $f_{i}\left(z_{i}\right)\leq \sum_{j=0}^{u_{i}-l_{i}}\lambda_{i,j}f_{i}\left(l_{i}+j\right)=\sum_{j=0}^{u_{i}-l_{i}}\lambda_{i,j}p_{i}^{l_{i}+j}$, and hence:

(14) $$\min\sum_{i=1}^{n}f_{i}\left(z_{i}\right)\leq \min\sum_{i=1}^{n}\sum_{j=0}^{u_{i}-l_{i}}\lambda_{i,j}p_{i}^{l_{i}+j}$$

Suppose that $z_{1}^{\prime },\ldots .,z_{n}^{\prime }$ are the optimal solutions to Equation (12); there must exist *n* integers $j_{1},\ldots .,j_{n}$, such that $z_{i}^{\prime }\in \left[l_{i}+j_{i},l_{i}+j_{i}+1\right]$. For any i∈{1,2,…,n}, let $\lambda_{i,j_{i}}^{\prime }=1+l_{i}+j_{i}-z_{i},\lambda_{i,j_{i}+1}^{\prime }=z_{i}^{\prime }-l_{i}-j_{i}$, and the others $\lambda_{i,j}^{\prime }s$ are zero. Obviously, $\lambda_{1,0}^{\prime },\lambda_{1,1}^{\prime },\ldots ,\lambda_{n,u_{n}-l_{n}}^{\prime }$ are feasible solutions of Equation (13). Based on the definition of $f_{i}\left(x\right)$, $f_{i}\left(z_{i}^{\prime }\right)=\left(z_{i}^{\prime }-l_{i}-j_{i}\right)\left(p_{i}^{l_{i}+j_{i}+1}-p_{i}^{l_{i}+j_{i}}\right)+p_{i}^{l_{i}+j_{i}}=\lambda_{i,j_{i}}^{\prime }p_{i}^{l_{i}+j_{i}}+\lambda_{i,j_{i}+1}^{\prime }p_{i}^{l_{i}+j_{i}+1}=\sum_{j=0}^{u_{i}-l_{i}}\lambda_{i,j}^{\prime }p_{i}^{l_{i}+j}$. Thus, we can have the following formula.

(15) $$\min\sum_{i=1}^{n}\sum_{j=0}^{u_{i}-l_{i}}\lambda_{i,j}p_{i}^{l_{i}+j}\leq \sum_{i=1}^{n}\sum_{j=0}^{u_{i}-l_{i}}\lambda_{i,j}^{\prime }p_{i}^{l_{i}+j}=\sum_{i=1}^{n}f_{i}\left(z_{i}^{\prime }\right)=\min\sum_{i=1}^{n}f_{i}\left(z_{i}\right)$$

In conclusion, optimization Equation (12) is equivalent to linear programming Equation (13). ☐

### 5.2. Linear Programming-Based Approximation Algorithm

Suppose that $\lambda_{1,0}^{\prime },\lambda_{1,1}^{\prime },\ldots ,\lambda_{n,u_{n}-l_{n}}^{\prime }$ are the optimal solutions to Equation (13). For any i∈{1,2,…,n}, let $z_{i}^{\prime }=\sum_{j=0}^{u_{i}-l_{i}}\lambda_{i,j}^{\prime }\left(l_{i}+j\right)$. Thus, $z_{1}^{\prime },z_{2}^{\prime },\ldots ,z_{n}^{\prime }$ are the optimal solutions to Equation (12) according to the proof of Theorem 8. To obtain the feasible solutions of integer optimization Equation (2), rounding optimal fractional solutions is a natural idea [29]. The following theorem guarantees that the ratio bound of the rounding approach is $1+p_{min}$, where $p_{min}$ is the minimum of the probabilities of one transmission failure along the given end-to-end path.

**Theorem** **9.**  *For any* i∈{1,2,…,n}, *the ratio bound of the rounding approach is* $1+p_{\min}$, *if* $\left\lfloor z_{i}^{\prime }\right\rfloor \geq \frac{\ln p_{\min}}{\ln p_{i}}$, *where* $p_{\min}=\min\left\{p_{1},p_{2},\ldots ,p_{n}\right\}$.

**Proof.**  Suppose that $z_{1}^{\prime },z_{2}^{\prime },\ldots ,z_{n}^{\prime }$ are the optimal solutions to Equation (12), which are derived from the optimal solutions of linear programming Equation (13). Denote $k_{1}^{\prime },k_{2}^{\prime },\ldots ,k_{n}^{\prime }$ as the optimal solutions to Equation (1). From Theorem 7, the approximation ratio *r* satisfies the following formula.

(16) $$r=\frac{\sum_{i=1}^{n}1-p_{i}^{k_{i}^{\prime }}}{\sum_{i=1}^{n}1-p_{i}^{\left\lfloor z_{i}^{\prime }\right\rfloor }}=\frac{n-\sum_{i=1}^{n}p_{i}^{k_{i}^{\prime }}}{n-\sum_{i=1}^{n}p_{i}^{\left\lfloor z_{i}^{\prime }\right\rfloor }}\leq \frac{n-\sum_{i=1}^{n}f_{i}\left(z_{i}^{\prime }\right)}{n-\sum_{i=1}^{n}p_{i}^{\left\lfloor z_{i}^{\prime }\right\rfloor }}$$

According to the proof of Theorem 8, for any i∈{1,2,…,n}, there must exist integer $j_{i}$ in $\left\{0,1,\ldots ,u_{i}-l_{i}-1\right\}$, such that $z_{i}^{\prime }\in \left[l_{i}+j_{i},l_{i}+j_{i}+1\right]$. Based on the convexity of $p_{i}^{x}$, we know that:

$$f_{i}\left(z_{i}^{\prime }\right)=\left(1+l_{i}+h-z_{i}^{\prime }\right)p_{i}^{l_{i}+h}+\left(z_{i}^{\prime }-l_{i}-h\right)p_{i}^{l_{i}+h+1}\geq p_{i}^{z_{i}^{\prime }}$$

Let $p_{\min}=\min\left\{p_{1},p_{2},..,p_{n}\right\}$; we have:

$$n-\sum_{i=1}^{n}f_{i}\left(z_{i}^{\prime }\right)\leq n-\sum_{i=1}^{n}p_{i}^{z_{i}^{\prime }}<n-\sum_{i=1}^{n}p_{i}^{\left\lfloor z_{i}^{\prime }\right\rfloor +1}\leq n-p_{\min}\sum_{i=1}^{n}p_{i}^{\left\lfloor z_{i}^{\prime }\right\rfloor }$$

Additionally, then, the approximation ratio *r* satisfies the following formula:

(17) $$\begin{array}{cc} r<\frac{n-p_{\min}\sum_{i=1}^{n}p_{i}^{\left\lfloor z_{i}^{\prime }\right\rfloor }}{n-\sum_{i=1}^{n}p_{i}^{\left\lfloor z_{i}^{\prime }\right\rfloor }} & =\frac{n-np_{\min}+np_{\min}-p_{\min}\sum_{i=1}^{n}p_{i}^{\left\lfloor z_{i}^{\prime }\right\rfloor }}{n-\sum_{i=1}^{n}p_{i}^{\left\lfloor z_{i}^{\prime }\right\rfloor }}\\ & =\frac{n-np_{\min}+p_{\min}\left(n-\sum_{i=1}^{n}p_{i}^{\left\lfloor z_{i}^{\prime }\right\rfloor }\right)}{n-\sum_{i=1}^{n}p_{i}^{\left\lfloor z_{i}^{\prime }\right\rfloor }}\\ & =\frac{n-\sum_{i=1}^{n}p_{\min}}{n-\sum_{i=1}^{n}p_{i}^{\left\lfloor z_{i}^{\prime }\right\rfloor }}+p_{\min} \end{array}$$

For any i∈{1,2,…,n}, since $\left\lfloor z_{i}^{\prime }\right\rfloor \geq \frac{\ln p_{\min}}{\ln p_{i}}$, $p_{i}^{\left\lfloor z_{i}^{\prime }\right\rfloor }\leq p_{\min}$ and $\frac{n-\sum_{i=1}^{n}p_{\min}}{n-\sum_{i=1}^{n}p_{i}^{\left\lfloor z_{i}^{\prime }\right\rfloor }}<1$. In conclusion, the ratio bound is $1+p_{\min}$. ☐

The link properties vary as a result of environmental conditions changing at a longer timescale [6,15], then the difference of failure transmission probability among links is little. Thus, the premises of Theorem 9 are always satisfied in practical sensor networks. Since the objective function value of Equation (12) is decreasing with respect to $z_{i}$, its optimum value can be achieved if and only if $\sum_{i=1}^{n}a_{i}z_{i}=\Delta$. For a general linear programming problem, finding optimal solutions incurs high computational overhead. However, it can be efficiently solved if the problem is to optimize a linear function subject to linear equality constraints [30].

The linear programming-based distributed approximation algorithm running at each sensor node is described in Algorithm 2, which is derived from the convex programming algorithm in [30]. $f_{h}^{L}\left(x\right)$ and $f_{h}^{R}\left(x\right)$ are the left and right derivative of $f_{h}\left(x\right)$, respectively. The proposed algorithm in [30] can yield optimal solutions for the piecewise linear convex function. For any i∈{1,2,…,n}, the derivative of $f_{i}\left(x\right)$ is discontinuous and constant. According to the proposed algorithm [30], the termination condition can be satisfied when the optimal retransmission threshold is not $L_{i}$ or $U_{i}$. The maximum number for iteration rounds can be assigned in advance. The algorithm can also be stopped if the convergence condition is not achieved. Therefore, figuring out the retransmission threshold of node $j_{i}$ can be achieved.

## 6. Lagrange Multiplier-Based Distributed *O*(1)-Approximation Algorithm

In practical sensor networks, the optimal retransmission thresholds hardly hit the given upper or lower bounds. Therefore, we aim at finding the optimal retransmission thresholds when the ranges of the upper and lower bounds of the retransmission thresholds are big enough. In Section 6.1, we formalize the problem as an integer optimization problem and provide a mathematical method for finding the approximate solution of the problem. Then, a Lagrange multiplier-based distributed O(1)-approximation algorithm with time complexity O(1) is provided.

### 6.1. Mathematical Foundations

Compared to integer optimization Equation (1), the problem of optimal retransmission thresholds in the case that the ranges of the upper and lower bounds of retransmission thresholds are big enough can be formulated as follows, which implies that the optimization problem has no interval constraints of retransmission thresholds.
**Algorithm 2:** Linear Programming-Based distributed Approximation Algorithm for the retransmission threshold (LPAA). 
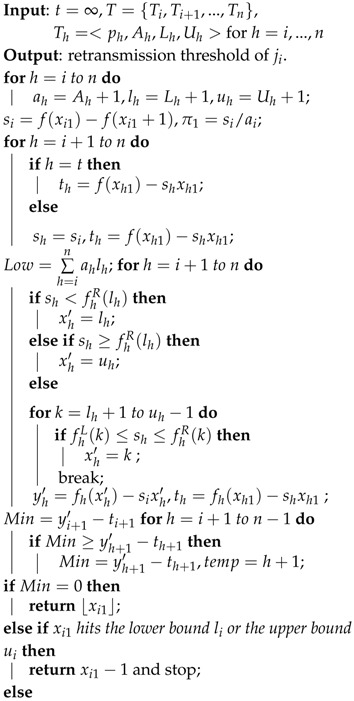
 According to the convex programming algorithm [30], adjust *x*_*i*1_ and *x_t_*;

(18) $$\begin{array}{cc} \max & \sum_{i=1}^{n}\left(1-p_{i}^{k_{i}}\right)\\ s.t. & \sum_{i=1}^{n}a_{i}k_{i}\leq \Delta \\ & k_{i}\in Z^{+},i\in \left\{1,2,\ldots ,n\right\} \end{array}$$

The following lemma provides a mathematical method to figure out the optimal solutions of Equation (18) in the real number field; that is for any *i*, $k_{i}$ is a real number. Lemma 10 is the foundation for the rounding technique.

**Lemma** **10.**  *Let* $\Lambda =\sum_{i=1}^{n}\left(1-p_{i}^{x_{i}}\right)+\omega \left(\sum_{i=1}^{n}a_{i}x_{i}-\Delta \right)$. *If there exist* $x_{1}^{\prime },x_{2}^{\prime },\ldots ,x_{n}^{\prime }$, *such that for any* i∈{1,2,…,n}, *the following equations hold:*

$$\frac{\partial \Lambda }{\partial x_{i}^{\prime }}=0$$

*then* $x_{1}^{\prime },\ldots ,x_{n}^{\prime }$ *are the optimal solutions to the following problem.*

(19) $$\begin{array}{cc} \max & \sum_{i=1}^{n}\left(1-p_{i}^{x_{i}}\right)\\ s.t. & \sum_{i=1}^{n}a_{i}x_{i}\leq \Delta \\ & x_{i}\in R,i\in \left\{1,2,\ldots ,n\right\} \end{array}$$

**Proof.**  For any i∈{1,2,…,n}, $p_{i}^{x}$ is a decreasing function. By contradiction, it is easily proven that the objective function achieves the maximum if and only if $\sum_{i=1}^{n}a_{i}x_{i}=\Delta$. Since $x_{1},x_{2},\ldots ,x_{n}$ are real numbers and the only constraint is equality, then we can derive the optimal solutions by the Lagrange multiplier method. We integrate the objective function and the constraint multiplied by *ω* to the following Lagrange function:

$$\Lambda \left(x_{1},x_{2},\ldots ,x_{n},\omega \right)=\sum_{i=1}^{n}\left(1-p_{i}^{x_{i}}\right)+\omega \left(\sum_{i=1}^{n}a_{i}x_{i}-\Delta \right)$$

Since $\sum_{i=1}^{n}a_{i}x_{i}-\Delta =0$, $\Lambda (x_{1},\ldots ,x_{n},\omega )$ is a function with respect to $x_{1},\ldots ,x_{n}$. The gradients on $x_{1},\ldots ,x_{n}$ and *ω* are as follows:

(20) $$\left\{\begin{array}{cc} \frac{\partial \Lambda }{\partial x_{1}} & =-p_{1}^{x_{1}}\ln\left(p_{1}\right)+\omega a_{1}\\ & \vdots \\ \frac{\partial \Lambda }{\partial x_{i}} & =-p_{i}^{x_{i}}\ln\left(p_{i}\right)+\omega a_{i}\\ & \vdots \\ \frac{\partial \Lambda }{\partial x_{n}} & =-p_{n}^{x_{n}}\ln\left(p_{n}\right)+\omega a_{n}\\ \frac{\partial \Lambda }{\partial \omega } & =\sum_{i=1}^{n}a_{i}x_{i}-\Delta \end{array}\right.$$

For any i∈{1,2,…,n} and j∈{1,2,…,n}, we have $\frac{\partial^{2}\Lambda }{\partial x_{i}\partial x_{j}}=0$ if i≠j. For any i∈{1,2,…,n}, it is easily derived that $\frac{\partial^{2}\Lambda }{\partial^{2}x_{i}}=-p_{i}^{x_{i}}\ln^{2}\left(p_{i}\right)$. The Hessian matrix of $\Lambda (x_{1},x_{2},\ldots ,x_{n})$ is as follows.

$$\frac{\partial^{2}\Lambda }{\partial x_{i}\partial x_{j}}=-\left(\begin{array}{ccccc} p_{1}^{x_{1}}\ln^{2}\left(p_{1}\right) & 0 & \ldots & \ldots & 0\\ 0 & \ddots & 0 & \cdots & \cdots \\ \ldots & 0 & p_{i}^{x_{i}}\ln^{2}\left(p_{i}\right) & 0 & \ldots \\ \cdots & \ldots & 0 & \ddots & 0\\ \ldots & \ldots & \ldots & 0 & p_{n}^{x_{n}}\ln^{2}\left(p_{n}\right) \end{array}\right)$$

Then, the following equalities can be easily derived.

$$\begin{array}{c} \lambda E-\frac{\partial^{2}\Lambda \left(x_{1},x_{2},\ldots ,x_{n}\right)}{\partial x_{i}\partial x_{j}}\\ =\left(\begin{array}{ccccc} \lambda +p_{1}^{x_{1}}\ln^{2}\left(p_{1}\right) & 0 & \ldots & \ldots & 0\\ 0 & \ddots & 0 & \cdots & \cdots \\ \ldots & 0 & \lambda +p_{i}^{x_{i}}\ln^{2}\left(p_{i}\right) & 0 & \ldots \\ \cdots & \ldots & 0 & \ddots & 0\\ \ldots & \ldots & \ldots & 0 & \lambda +p_{n}^{x_{n}}\ln^{2}\left(p_{n}\right) \end{array}\right)\\ \left|\lambda E-\frac{\partial^{2}\Lambda \left(x_{1},x_{2},\ldots ,x_{n}\right)}{\partial x_{i}\partial x_{j}}\right|=\prod_{i=1}^{n}\left(\lambda +p_{i}^{x_{i}}\ln^{2}\left(p_{i}\right)\right) \end{array}$$

Since all of the eigenvalues of $\frac{\partial^{2}\Lambda \left(x_{1},\ldots ,x_{n}\right)}{\partial x_{i}\partial x_{j}}$ are negative, the Hessian matrix of $\Lambda (x_{1},\ldots ,x_{n})$ is negative definite. In conclusion, if there exist $x_{1}^{\prime },\ldots ,x_{n}^{\prime }$, such that for any i∈{1,2,…,n}, $\frac{\partial \Lambda }{\partial x_{i}^{\prime }}=0$, then $x_{1}^{\prime },\ldots ,x_{n}^{\prime }$ are the optimal solutions to Equation (19). ☐

**Theorem** **11.**  $x_{1}^{\prime },\ldots ,x_{n}^{\prime }$ *are the optimal solutions to Equation (19), if for any* i∈{1,2,…,n}, $x_{i}^{\prime }$ *satisfies Equation (21).*

(21) $$x_{i}^{\prime }=\frac{\Delta -\sum_{i=1}^{n}a_{i}\log_{p_{i}}^{-a_{i}{\left(\ln p_{i}\right)}^{-1}}}{\ln p_{i}\sum_{i=1}^{n}a_{i}{\left(\ln p_{i}\right)}^{-1}}+\log_{p_{i}}^{-a_{i}{\left(\ln p_{i}\right)}^{-1}}$$

**Proof.**  According to the proof of Lemma 10, if $x_{1}^{\prime },\ldots ,x_{n}^{\prime }$ are the solutions of Equation (20), then they are the optimal solutions to Equation (19). If $\frac{\partial \Lambda }{\partial x_{i}^{\prime }}=0$, we have $p_{i}^{x_{i}^{\prime }}\ln\left(p_{i}\right)=\omega a_{i}$. For any i∈{1,2,…,n}, since $a_{i}>0,0<p_{i}<1$ and ω<0, we have that:

$$x_{i}^{\prime }=\log_{p_{i}}^{\omega a_{i}{\left(\ln p_{i}\right)}^{-1}}=\log_{p_{i}}^{-\omega }+\log_{p_{i}}^{-a_{i}{\left(\ln p_{i}\right)}^{-1}}$$

Since $\frac{\partial \Lambda }{\partial \omega }=\sum_{i=1}^{n}a_{i}x_{i}^{\prime }-\Delta =0$, it is easily derived that:

$$\sum_{i=1}^{n}a_{i}\left(\log_{p_{i}}^{-\omega }+\log_{p_{i}}^{-a_{i}{\left(\ln p_{i}\right)}^{-1}}\right)=\Delta$$

By simple calculation, we can get the following equations:

$$\sum_{i=1}^{n}\frac{a_{i}\ln\left(-\omega \right)}{\ln p_{i}}+\sum_{i=1}^{n}a_{i}\log_{p_{i}}^{-a_{i}{\left(\ln p_{i}\right)}^{-1}}=\Delta$$

$$\ln\left(-\omega \right)=\frac{\Delta -\sum_{i=1}^{n}a_{i}\log_{p_{i}}^{-a_{i}{\left(\ln p_{i}\right)}^{-1}}}{\sum_{i=1}^{n}a_{i}{\left(\ln p_{i}\right)}^{-1}}$$

In conclusion, $x_{1}^{\prime },\ldots ,x_{n}^{\prime }$ are the optimal solutions to Equation (19), if for any i∈{1,2,…,n}, $x_{i}^{\prime }$ satisfies Equation (21). ☐

It is obvious that $\log_{p_{i}}^{-a_{i}{\left(\ln p_{i}\right)}^{-1}}=\frac{\ln a_{i}+\ln\left(-{\left(\ln p_{i}\right)}^{-1}\right)}{\ln p_{i}}$; the following equation can be derived.

(22) $$x_{i}^{\prime }=\frac{\Delta -\sum_{i=1}^{n}a_{i}\left(\frac{\ln a_{i}+\ln\left(-{\left(\ln p_{i}\right)}^{-1}\right)}{\ln p_{i}}\right)}{\ln p_{i}\sum_{i=1}^{n}a_{i}{\left(\ln p_{i}\right)}^{-1}}+\frac{\ln a_{i}+\ln\left(-{\left(\ln p_{i}\right)}^{-1}\right)}{\ln p_{i}}$$

### 6.2. Lagrange Multiplier-Based Distributed O(1)-Approximation Algorithm

The computation of the logarithm is undesired to execute on the sensor node; the log values needed can be stored on the sensor node in advance. For example, three arrays a[99],b[99] and c[99] are forwarded to the sensor node at first, where $a\left[h\right]=\ln h,b\left[h\right]=\ln\left(0.01h\right),c\left[h\right]=\ln\left(-{\left(\ln0.01h\right)}^{-1}\right)$ and h∈{1,2,…,99}. Then, the log values needed by Equation (22) are stored in the sensor node. The proposed Lagrange multiplier-based distributed approximation algorithm is described in Algorithm 3. It is easily known that the computation cost is O(n), where *n* is the number of hops from the source node to the destination node. Since the number of sensor nodes along the given end-to-end delivery path is no more than a few dozen, hence the computation complexity of the algorithm is O(1).
**Algorithm 3:** Lagrange Multiplier-based Distributed Approximation Algorithm for the retransmission threshold (LMDAA). 
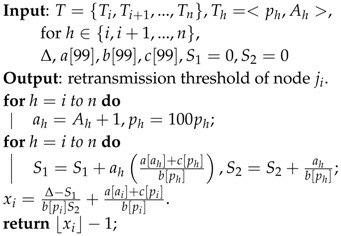



Similarly to the linear programming-based approximation algorithm, approximate solutions to integer optimization Equation (18) can be derived by rounding the optimal fractional solutions. The following theorem guarantees that the ratio bound of the rounding approach is $1+p_{min}$. Due to $0<p_{min}<1$, the ratio bound of the Lagrange multiplier-based approximation algorithm is two.

**Theorem** **12.**  *Suppose that* $x_{1}^{\prime },x_{2}^{\prime },\ldots ,x_{n}^{\prime }$ *are the optimal solutions to Equation (19), then* $\left\lfloor x_{1}^{\prime }\right\rfloor ,\ldots ,\left\lfloor x_{n}^{\prime }\right\rfloor$ *are the feasible solutions to Equation (18). The ratio bound is* $1+p_{min}$, *if for any* i∈{1,2,…,n}, $\left\lfloor x_{i}^{\prime }\right\rfloor \geq \frac{\ln p_{\min}}{\ln p_{i}}$, *where* $p_{\min}=\min\left\{p_{1},p_{2},\ldots ,p_{n}\right\}$.

**Proof.**  Since $x_{1}^{\prime },x_{2}^{\prime },\ldots ,x_{n}^{\prime }$ are the optimal solutions to Equation (19), $\sum_{i=1}^{n}a_{i}x_{i}^{\prime }=\Delta$, and hence, $\sum_{i=1}^{n}a_{i}\left\lfloor x_{i}^{\prime }\right\rfloor \leq \Delta$. Thus, $\left\lfloor x_{1}^{\prime }\right\rfloor ,\ldots ,\left\lfloor x_{n}^{\prime }\right\rfloor$ are the feasible solutions to Equation (18). Suppose that $k_{1}^{\prime },k_{2}^{\prime },\ldots ,k_{n}^{\prime }$ are the optimal solutions to Equation (18). Obviously, the objective function value of Equation (19) is an upper bound of that of Equation (18). Thus, the following inequalities can be derived.

$$\sum_{i=1}^{n}\left(1-p_{i}^{k_{i}^{\prime }}\right)\leq \sum_{i=1}^{n}\left(1-p_{i}^{x_{i}^{\prime }}\right)<\sum_{i=1}^{n}\left(1-p_{i}^{\left\lfloor x_{i}^{\prime }\right\rfloor +1}\right)$$

Let $p_{\min}=\min\left\{p_{1},p_{2},\ldots ,p_{n}\right\}$. Thus, $\sum_{i=1}^{n}\left(1-p_{i}^{k_{i}^{\prime }}\right)<\sum_{i=1}^{n}\left(1-p_{\min}p_{i}^{\left\lfloor x_{i}^{\prime }\right\rfloor }\right)$, and hence, the approximation ratio *r* satisfies the following equation:

$$r=\frac{\sum_{i=1}^{n}\left(1-p_{i}^{k_{i}^{\prime }}\right)}{\sum_{i=1}^{n}\left(1-p_{i}^{\left\lfloor x_{i}^{\prime }\right\rfloor }\right)}<\frac{\sum_{i=1}^{n}\left(1-p_{\min}p_{i}^{\left\lfloor x_{i}^{\prime }\right\rfloor }\right)}{\sum_{i=1}^{n}\left(1-p_{i}^{\left\lfloor x_{i}^{\prime }\right\rfloor }\right)}=\frac{n-np_{\min}}{n-\sum_{i=1}^{n}p_{i}^{\left\lfloor x_{i}^{\prime }\right\rfloor }}+p_{\min}$$

In conclusion, the ratio bound is $1+p_{min}$, if for any i∈{1,2,…,n}, $\left\lfloor x_{i}^{\prime }\right\rfloor \geq \frac{\ln p_{\min}}{\ln p_{i}}$. Similarly, we know that the premises of Theorem 12 are easily satisfied in practical sensor networks. ☐

## 7. Experiment Evaluation

The effectiveness and efficiency of the proposed algorithms are evaluated through simulations in this section. Several experiments are conducted to demonstrate the relationships between the performance of real-time data delivery and the input parameter, such as deadline, the number of hops of the given delivery path and the number of packets queued at the relay node. The first two group of simulations are carried out by MATLAB, and the third group is implemented with NS2.

One hundred sensor nodes and the sink are randomly deployed into a region of size 200 m × 200 m (m for meters), and we assume that the sensors have the same transmission radius. In each simulation, source and destination nodes are randomly selected. Each simulation is repeated 100 times and the simulation result corresponds to the average value over 100 times. To understand the benefits of the proposed algorithms, the comparison with the general method is conducted. The main idea of the general method is that we set the same retransmission threshold for all of the sensor nodes.

**Figure 3:**
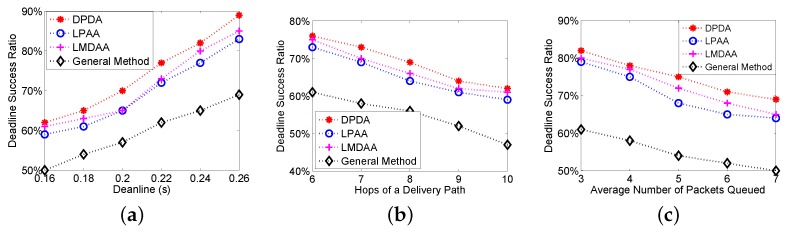
Deadline Success Ratio (DSR) of the proposed algorithms. (**a**) DSR *vs.* deadlines; (**b**) DSR *vs.* the number of hops; (**c**) DSR *vs*. the average number of packets queued.

The first group of experiments is to investigate the Deadline Success Ratio (DSR) of the proposed algorithms, where DSR is the ratio of the packets delivered to the destination before their deadlines. Figure 3a shows the relationship between the deadlines and DSRs. The proposed algorithms have better performance. For example, when the deadline is 0.22 s, the DSRs of LMDAA and general method are 74% and 61%, respectively. Furthermore, we investigate the impact of the number of hops of a delivery path on the DSR. The remaining time to the deadline and link quality have been considered; thus, our algorithms can achieve higher deadline success ratios as shown in Figure 3b. Figure 3c depicts the relationship between the average number of packets queued at relay nodes and DSRs. From Figure 3c, the DSR becomes worse with the increase of the packets queued on the condition that the deadline is a constant. LMDAA is a distributed algorithm and enables the node to adaptively set the optimal retransmission threshold based on the link quality and the remaining time to the deadline. Therefore, LMDAA has better performance in terms of DSR than that of LPAA.

**Figure 4:**
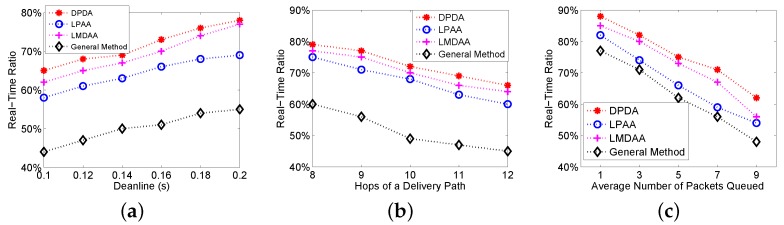
Real-Time Ratio (RTR) of the proposed algorithms. (**a**) RTR *vs.* deadlines; (**b**) RTR *vs.* number of hops; (**c**) RTR *vs.* the average number of packets Queued.

The second group of experiments is to investigate the Real-Time Ratio (RTR) of the proposed algorithms, where RTR is the ratio of the packets delivered to the destination before their deadlines among the packets successfully delivered to the destination node. Figure 4a shows the relationship between the deadlines and RTRs. As expected, the proposed algorithms can reach higher RTR than that of the general method. Figure 4b depicts the relationship between the number of hops of a delivery path and RTRs. The figure shows that our algorithms have better performance. For example, the real-time ratio of LPAA is more than 60%. Similarly, we investigate the impact of the average number of packets queued at relay nodes on RTR, with results illustrated in Figure 4c. Experimental results show that the proposed algorithms can reduce the deliveries of the packets, which cannot meet their deadlines. Therefore, our algorithms can improve the real-time ratio and energy efficiency.

**Figure 5:**
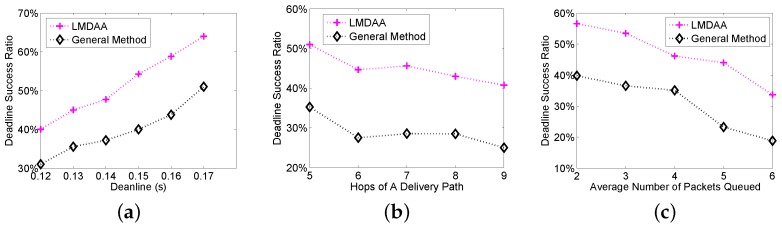
Deadline success ratio by NS2 simulation. (**a**) DSR *vs.* deadlines; (**b**) DSR *vs.* the number of hops; (**c**) DSR *vs.* the average number of packets queued.

The third group of simulations is implemented with NS2, which is a widely-used simulation tool in wireless sensor networks. From the experimental results of the first two groups of simulations, LMDAA can reveal the effectiveness of the proposed algorithms more neutrally, then we compare the results of LMDAA and the general method. The reasons why DSRs in the simulations implemented by NS2 are lower than those in case of MATLAB are as follows. For identical transmission failure probability, the link quality in NS2 simulation is much worse, which leads to a lower packet delivery ratio. Secondly, we have to set stationary retransmission thresholds in advance in NS2 simulations, and the retransmission thresholds cannot be adaptively set based on the link quality and remaining time during the packet delivery. As shown in Figure 5a–c, the proposed algorithm has better performance in real-time data delivery, in the case of worse wireless links or a severe delivery delay requirement.

**Figure 6:**
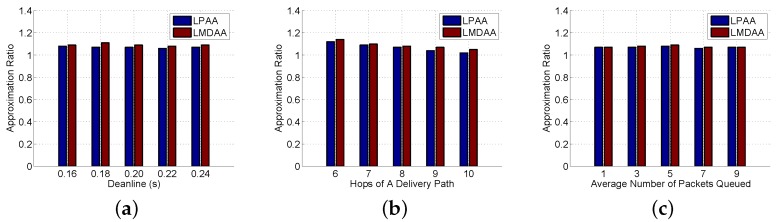
Computing performance of Linear Programming-based (1 + *p_min_*)-Approximation Algorithm (LPAA) and Lagrange Multiplier-based Distributed Approximation Algorithm (LMDAA). (**a**) Approximation Ratio (AR) *vs.* deadlines; (**b**) AR *vs.* the number of hops; (**c**) AR *vs.* the average number of packets queued.

The forth group of experiments is to investigate the computing performance of LPAA and LMDAA, and the correctness of the approximation ratio is verified. We prove that the objective function value of Equation (12) is a lower bound of that of Equation (2), then $\sum_{i=1}^{n}\left(1-f_{i}\left(z_{i}\right)\right)$ generated by the optimal solution of Equation (12) is an upper bound of Equation (1). In the experiments, the Approximation Ratio (AR) is the ratio of $\sum_{i=1}^{n}\left(1-f_{i}\left(z_{i}\right)\right)$ generated by the optimal solutions of linear programming Equation (13) to that of the approximated solutions output by LPAA and LMDAA. Figure 6a–c demonstrates the relationships between the approximation ratio and the input parameter. Experimental results show that the objective function value of the approximation results returned by LPAA and LMDAA is very close to that of the optimal ones. Additionally, the proposed approximation algorithms can achieve high accuracy in terms of optimal retransmission thresholds.

## 8. Conclusions

The retransmission threshold in wireless sensor networks is critical to the latency of data transmitting in the networks. The problem of finding optimal retransmission thresholds for each node along a delivery path is defined and is formalized as an integer optimization problem. A dynamic programming-based distributed algorithm for finding the optimal retransmission threshold is proposed. The correctness of the algorithm is proven, and its time and space complexity are analyzed. When the delivery delay Δ is greater than polynomial, a linear programming-based $\left(1+p_{min}\right)$-approximation algorithm is proposed. Furthermore, in the case of the ranges of the upper and lower bounds of the retransmission thresholds being big enough, a Lagrange multiplier-based distributed O(1)-approximation algorithm with time complexity O(1) is proposed. Simulation results show that the proposed algorithms have better performance for real-time data delivery.
